# Predictors of Bilateral SLN Radiocolloid Detection in Endometrial Carcinoma

**DOI:** 10.1055/s-0043-1777693

**Published:** 2023-12-26

**Authors:** Anamarija Jankulovska, Sasho Stojcevski, Igor Aluloski, Mile Tanturovski, Nevena Manevska, Ana Daneva Markova, Sinisa Stojanoski

**Affiliations:** 1 Institute of Pathophysiology and Nuclear Medicine “Acad Isac S. Tadzer”, Faculty of Medicine, University of “Ss. Cyril and Methodius”, Skopje, Republic of North Macedonia; 2 University Clinic for Obstetrics and Gynecology, Skopje, Republic of North Macedonia

**Keywords:** endometrial carcinoma, sentinel lymph node biopsy, risk factors, radiocolloid, bilateral lymphatic drainage

## Abstract

**Introduction** Sentinel lymph node (SLN) mapping is an alternative method to conventional lymphadenectomy for nodal status assessment in patients with stage I/II endometrial carcinoma (EC). This study aimed to analyze the potential predictors of unsuccessful bilateral detection of SLN after the application of radiocolloid in EC.

**Materials and Methods** A prospective, observational, cross-sectional study was performed on 41 patients with EC in preoperative stage I, who underwent SLN mapping after cervical application of 4mCi ^99m^ Tc-SENTI-SCINT. The demographic, clinical, and tumor-related data were obtained from the patient's medical records. Univariate analysis was used to analyze the potential factors associated with an unsuccessful bilateral SLN biopsy.

**Results** The bilateral SLN detection rate of planar lymphoscintigraphy, single photon emission computed tomography/computed tomography, and gamma probe was 29.26, 41.46, and 26.82%, correspondingly. None of the 16 analyzed risk factors showed statistical significance for nonconclusive bilateral SLN biopsy.

**Conclusion** Larger scale studies are needed to determine the exact risk factors for unsuccessful bilateral mapping of the lymphatic drainage after cervical application of the radiotracers. This will eventually lead to improvement in bilateral SLN detection in EC patients, so unilateral lymphadenectomy could be avoided.

## Introduction

Sentinel lymph node (SLN) biopsy is a successful alternative to the conventional elective lymphadenectomy for the evaluation of the nodal status in patients with endometrial carcinoma (EC), who were preoperatively staged as International Federation of Gynecology and Obstetrics (FIGO) 1 or 2. 1 The technique was first reported by Cabanas in penis carcinoma in 1977, 2 while introduced by Burke et al for EC in 1996. 3 Colorimetric, radionuclide, and fluorescent methods are currently the most commonly used techniques for SLN detection. 4

After interstitial application, the radiocolloid enters the lymphatic capillaries and through the lymph vessels reaches the first drainage lymph node, where it remains trapped by the macrophages, unlike other tracers that are characterized by rapid lymphatic clearance. This provides a unique opportunity for preoperative localization of the SLN on a planar lymphoscintigraphy and single photon emission computed tomography/computed tomography (SPECT/CT), by which significantly eases the SLN detection, thus reducing the time duration of the surgical intervention. 5 6

The uterus is a central organ with bilateral lymphatic drainage. This means that it is necessary to remove at least two SLNs, one in each hemipelvis. According to studies that treated each hemipelvis as a separate unit, a SLN algorithm was proposed that would increase the accuracy and sensitivity of the method. If SLN has not been detected in any hemipelvis, a site-specific lymphadenectomy should be performed. 7

Taking this into consideration, bilateral SLN mapping on scintigraphic images and bilateral SLN biopsy is very important. Hence, performing a unilateral lymphadenectomy would be avoided, and thus the risk of complications, such as lymphedema/lymphocytes which increases with the number of removed lymph nodes, would be reduced. 8

The purpose of this study was to evaluate the impact of demographic, clinical, and tumor-related factors on the unsuccessful bilateral detection of SLN by cervical application of ^99m^ Tc-SENTI-SCINT as a tracer, in EC patients.

## Materials and Methods

In a prospective, observational, cross-sectional study, data from patients with preoperative first-stage EC who went through SLN mapping after application of ^99m^ Tc-SENTI-SCINT were analyzed. All patients over 18 years of age who were examined at the University Clinic of Gynecology and Obstetrics in Skopje with a diagnosis of EC in presumed FIGO I stage and who did not have contraindications for application of radiocolloid/surgery were offered the procedure for SLN biopsy and participation in the study. All patients who signed an informed consent in the period from 2018 to 2021 year, were included in the analysis. The study was approved by the Ethics Committee of the Medical Faculty in Skopje (approval number: 03-366/8).

### SLN Mapping

The tracer was applied cervically in four quadrants (4mCi), followed by SLN mapping on a dynamic study after application of the radiopharmaceutical and static images at 30 minutes, 60 minutes, and 120 minutes in the anteroposterior position (600 seconds/image, matrix 256 * 256 * 16). The planar images were taken with a Mediso DHV Nucline Spirit gamma camera.SPECT/CT images were taken after 120 to 180 minutes with OPTIMA NM/CT 640 GE Healthcare dual detector/4 slice CT camera: SPECT (60 projections for 15 seconds per projection, projection angle: 6 degrees, detector angle: 180 degrees, matrix 128 * 128) and CT (matrix 512 * 512, rotation time: 1 second, cross-sectional thickness: 2.5 mm, cross-sectional distance 2.5 mm).

### SLN Biopsy

The surgical method was laparotomy. SLN detection was made intraoperatively with a hand-held gamma probe (EUROPROBE SYSTEM III) based on the results of the preoperative scintigraphic mapping. In case of no SLN being found in any half of the pelvis, a site-specific lymph node dissection was required to assess unilateral lymph node status. In patients who were preoperatively assessed with high risk according to the ESMO-ESGO-ESTRO guidelines, after the SLN biopsy, a pelvic lymphadenectomy was performed according to the standard protocols. All patients underwent abdominal hysterectomy with bilateral salpingo-oophorectomy. In this regard, a metastatic SLN would lead to upstaging without the need for a radical lymphadenectomy, while metastasis-free detected SLN would avoid unnecessary lymphadenectomy in low and intermediate-risk patients.

### Pathohistological Analyses

All lymph nodes were standardly stained with hematoxylin and eosin for microscopic analysis. Additional immunohistochemical staining using a monoclonal antibody CKAE1/AE3 was used for enhanced analysis of those SLNs where no metastatic deposits were found at the initial cross-section.

## Statistical Analysis

Anamnestic data for the demographic characteristics and data from the medical records: age, body mass index (BMI), menarche, number of births, menopause, usage of estrogen therapy, smoking, comorbidity (diabetes, cardiovascular disease), family history for malignancy were taken from all patients. Tumor data were obtained postoperatively from the pathohistological report.

Statistical analysis of the data obtained from the research was performed in the statistical program SPSS 23.0. Non-parametric and parametric tests for independent samples (Fisher's exact test, chi-square test, Student's *t* -test) were used to compare the groups with unsuccessful (patients with unilateral SLN detection or undetected SLN in the pelvis) and successful bilateral SLN detection.

The correlations of age and BMI with the number of SLNs were analyzed with the Spearman correlation coefficient (R).

Statistical significance was defined at the level of *p* -value less than 0.05.

## Results

In the analyzed period, 41 patients were scheduled for a SLN biopsy. Of these, 39 patients had a successful detection of SLN in the pelvic region with a gamma probe. The bilateral detection rate for planar lymphoscintigraphy and SPECT/CT was 29.26% (in 12 patients) and 41.46% (in 17 patients), correspondingly, while intraoperative successful bilateral SLN biopsy was noted in only 11 patients (26.82%).

The mean age of the patients and the mean value of the BMI index are shown in Table 1 . Patients with successful and unsuccessful bilateral detection of SLN on planar lymphoscintigraphy, SPECT/CT, and intraoperatively, did not differ significantly, regarding age and BMI ( *p* > 0.05).

**Table 1:** Univariate analysis of age and BMI with successful SLN detection

SLN detection		Statistical parameter		*p* -Value level
		Mean ± SD	Min–max	
**Age (years)**				
SLN detection on planar lymphoscintigraphy	Unsuccessful bilateral	60.0 ± 7.3	47–72	*t* = 0.14, *p* = 0.89 ns
	Successful bilateral	59.9 ± 10.3	45–74	
SLN detection on SPECT/CT	Unsuccessful bilateral	61.6 ± 7.3	47–72	*t* = 1.16, *p* = 0.25 ns
	Successful bilateral	58.7 ± 8.7	45–74	
SLN detection intraoperatively	Unsuccessful bilateral	60.3 ± 7.1	46–72	*t* = 0.27, *p* = 0.78 ns
	Successful bilateral	59.5 ± 9.8	45–74	
** BМI (kg/m ^2^ ) **				
SLN detection on planar lymphoscintigraphy	Unsuccessful bilateral	32.32 ± 4.7	23.4–41.5	*t* = 0.67, *p* = 0.51 ns
	Successful bilateral	33.94 ± 8.5	25.2–53.3	
SLN detection on SPECT/CT	Unsuccessful bilateral	31.61 ± 4.2	23.4–39.9	*t* = 1.06, *p* = 0.29 ns
	Successful bilateral	33.66 ± 7.5	25.2–53.3	
SLN detection intraoperatively	Unsuccessful bilateral	32.26 ± 4.3	23.4–41.5	*t* = 0.61, *p* = 0.55 ns
	Successful bilateral	33.55 ± 8.6	25.2–53.3	

Abbreviations: BMI, body mass index; SD, standard deviation; SLN, sentinel lymph node; SPECT/CT, single-photon emission computed tomography/computed tomography.

Patients with bilateral SLN detection had a lower mean age compared to patients with unsuccessful bilateral detection, on planar lymphoscintigraphy, SPECT/CT, and intraoperatively (59.9 ± 10.3 years vs. 60.0 ± 7.3 years, *p* = 0.89, 58.7 ± 8.7 years vs. 61.6 ± 7.3 years, *p* = 0.25, and 59.5 ± 9.8 years vs. 60.3 ± 7.1 years, *p* = 0.78, respectively).

BMI had an insignificantly greater average value in patients with bilateral detection of SLN compared to patients with unsuccessful bilateral detection on planar lymphoscintigraphy, SPECT/CT and intraoperatively (33.94 ± 8.5kg/m ^2^ vs. 32.32 ± 4.7 kg/m ^2^ , *p* = 0.51, 33.66 ± 7.5 kg/m ^2^ vs. 31.61 ± 4.2 kg/m ^2^ , *p* = 0.29, and 33.55 ± 8.6 kg/m ^2^ vs. 32.26 ± 4.3 kg/m ^2^ , *p* = 0.55, respectively).

The age of the patients and the BMI were insignificantly correlated with the number of SLNs on planar lymphoscintigraphy, SPECT/CT and intraoperatively ( Table 2 ).

**Table 2:** Correlation between the age and BMI with the number of SLN and the time of visualization of SLN on scintigraphic images after radiocolloid application

Correlation	Spearman R	*p* -Value level
Age with the number of SLN on planar lymphoscintigraphy	− 0.12	0.45
BMI with number of SLN on planar lymphoscintigraphy	− 0.211	0.2
Age with the number of SLN on SPECT/CT	− 0.197	0.22
BMI with number of SLN on SPECT/CT	0.185	0.26
Age with the time of appearance of SLN on the image after application of radiocolloid	0.301	0.059
BMI with the time of the appearance of the SLN on the image after the application of radiocolloid	− 0.143	0.4

Abbreviations: BMI, body mass index; SLN, sentinel lymph node; SPECT/CT, single-photon emission computed tomography/computed tomography.

The total number of intraoperatively detected SLNs was 64. Eleven SLNs were detected only in the left hemipelvis, 17 in the right hemipelvis, while the rest were detected bilaterally. Only 11 patients underwent successful bilateral SLN biopsy (26.82%; Table 3 ).

**Table 3:** Distribution of patients regarding the total removed SLN, separately in the left hemipelvis, the right hemipelvis, and bilaterally

Number of removed SLN	Total, *n* (%)	Left hemipelvis, *n* (%)	Right hemipelvis, *n* (%)	Bilaterally, *n* (%)
0	0	0	0	0
1	22 (53.66)	9 (81.82)	13 (76.47)	0
2	10 (24.39)	0	3 (17.63)	7 (63.64)
3	6 (14.63)	1 (9.09)	1 (5.88)	4 (36.36)
4	1 (9.09)	1 (9.09)	0	0
Total	39	11	17	11

Abbreviation: SLN, sentinel lymph node.

The results of the statistical analysis did not confirm a significant difference between patients with unsuccessful and successful bilateral SLN detection regarding the clinically analyzed risk factors: age, BMI, number of births, menarche, menopause, estrogen therapy, diabetes, smoking, hypertension, other cardiovascular diseases, family history of malignancy, and tumor-related risk factors: tumor size, tumor histology, degree of differentiation, lymphovascular and myometrial invasion.

The results of the univariate analysis of demographic and clinical data of the patients with successful bilateral SLN biopsy are shown in Table 4 , while the results of the univariate analysis of tumor-related risk factors of the patients with successful bilateral SLN biopsy are shown in Table 5 .

**Table 4:** Univariate analysis of demographic and clinical data of the patients with successful bilateral SLN biopsy

Variable		Bilateral detection of SLN			*p* -Value level
		*n*	Unsuccessful, *n* = 30 (%)	Successful, *n* = 11 (%)	
Age (years)	< 60	20	13 (65.00)	7 (35.00)	X ^2 ^ = 0.64 *p* = 0.42 ns
	≥ 60	21	17 (80.95)	4 (19.05)	
BMI (kg/m ^2^ )	< 30	14	9 (64.28)	5 (35.72)	X ^2 ^ = 0.30 *p* = 0.58 ns
	≥ 30	27	21 (77.78)	6 (22.22)	
Number of births	0	4	1 (25.00)	3 (75.00)	Fisher's exact test *p* = 0.05 ns
	≥ 1	37	29 (78.37)	8 (21.62)	
Menarche	< 12 years	16	13 (81.25)	3 (18.75)	X ^2 ^ = 0.33 *p* = 0.56 ns
	> 12 years	25	17 (68.00)	8 (32.00)	
Menopause	Before 50 years	14	10 (71.43)	4 (28.57)	X ^2 ^ = 0.03 *p* = 0.85 ns
	After 50 years	27	20 (74.07)	7 (25.93)	
Estrogen therapy	Yes	3	2 (66.67)	1 (33.33)	X ^2 ^ = 0.17 *p* = 0.67 ns
	No	38	28 (73.68)	10 (26.32)	
Diabetes mellitus	Yes	14	10 (71.43)	4 (28.57)	X ^2 ^ = 0.03 *p* = 0.85 ns
	No	27	20 (74.07)	7 (25.93)	
Smoking	Yes	7	4 (57.14)	3 (42.86)	X ^2 ^ = 0.34 *p* = 0.56 ns
	No	34	26 (76.47)	8 (23.53)	
Hypertension	Yes	32	22 (68.75)	10 (31.25)	X ^2 ^ = 0.60 *p* = 0.44 ns
	No	9	8 (88.89)	1 (11.11)	
Other cardiovascular diseases	Yes	17	12 (70.59)	5 (29.41)	X ^2 ^ = 0.0019 *p* = 0.96 ns
	No	24	18 (75.00)	6 (25.00)	
Family history of malignancy	Yes	4	4 (100)	0 (0)	Fisher's exact test *p* = 0.55 ns
	No	37	26 (70.27)	11 (29.73)	

Abbreviations: BMI, body mass index; SLN, sentinel lymph node.

**Table 5:** Univariate analysis of tumor-related data in patients with successful bilateral SLN biopsy

Variable		Bilateral detection of SLN			*p* -Value level
		*n*	Unsuccessful, *n* = 30 (%)	Successful, *n* = 11 (%)	
Size of the tumor (cm)	< 2	17	15 (88.24)	2 (11.76)	X ^2 ^ = 2.17 *p* = 0.14 ns
	≥ 2	24	15 (62.50)	9 (37.50)	
Postoperative histology	Endometrial type	34	24 (70.59)	10 (29.41)	Fisher's exact test *p* = 0.65 ns
	Nonendometrial type	7	6 (85.71)	1 (14.29)	
Postoperative grade	Grade 1	6	5 (83.33)	1 (16.67)	X ^2 ^ = 0.44 *p* = 0.80 ns
	Grade 2	27	19 (70.37)	8 (29.63)	
	Grade 3	8	6 (75.00)	2 (25.00)	
Lymphovascular space invasion	Present	11	8 (72.73)	3 (27.27)	X ^2 ^ = 0.13 *p* = 0.72 ns
	Absent	30	22 (73.33)	8 (26.67)	
Myometrial invasion	< 50%	32	23 (71.88)	9 (28.12)	X ^2 ^ = 0.0053 *p* = 0.94 ns
	> 50%	11	7 (77.78)	4 (22.22)	

Abbreviation: SLN, sentinel lymph node.

## Discussion

The bilateral SLN detection rate in the EC differs, depending on the technique used, so the lowest rate has been reported when using a single tracer—44% (95% confidence interval: 38–50%) for blue dye. 9 Although the overall detection rate is similar for indocyanine green (ISG) and the dual-tracer method (blue dye and radiocolloid), the bilateral detection rate is higher for ISG (84.1% vs. 73.5%). 10 The superiority of ISG has been demonstrated by visualizing both lymph vessels and SLNs more clearly and more prominently through the visceral and retroperitoneal fat as well as rapid tracer entry into the SLNs. However, its usage has been limited by the need for a specific near-infrared optical system for detection. 11 In addition, the detection rate could be increased up to 96% when tracer reinjection is used. 12 Concerning the site of tracer application, cervical administration shows the highest bilateral pelvic detection rate. 13 14 Precise application of the tracer by the clinician is crucial for a good display of lymphatic drainage. When administering a radiocolloid, it is important that a large amount of preparation does not enter the bloodstream, because high activity in the bone marrow, liver, and spleen has been shown to be a significant factor for failed detection of the SLN on SPECT/CT. 15 16 On the other hand, this would reduce the tracer volume that needs to enter the lymph vessels and the activity at the injection site, which in the Sahbai et al study was associated with a lower detection rate. For optimal results, they proposed quantitative SPECT/CT analysis and possible tracer reinjection in patients with low activity at the site of application. 16 An additional factor for low detection rate on SPECT/CT and gamma probe is high abdominal/peritoneal activity. 15

Patient characteristics such as age and body weight could affect the success of the procedure through changes in the lymph vessels and lymphatic drainage itself. Angeles et al showed that the higher volume of radiotracer, patients under 75 years, and the tumor size below 2 cm were associated with a higher rate of preoperative detection of SLN after transvaginal echo-guided myometrial application of the tracer. 17 Similar results have been reported in other studies where advanced age was associated with failure of the method. Kraft and Havel in their study of 550 patients concluded that the lower average age was associated with higher number of SLNs on SPECT/CT compared to planar lymphoscintigraphy, while BMI had no statistically significant effect. 18 The results on the impact of BMI are also contradictory. According to a study by Eriksson et al, an increase in BMI was associated with decreased bilateral SLN biopsy. 19

In our study, we found no statistically significant impact of age and BMI on bilateral lymph node detection, though successful bilateral SLN mapping was associated with a lower mean age that correlates with the above-mentioned studies.

The intraoperative SLN detection rate is closely related to the surgeon's experience. A learning curve of at least 30 cases is required to achieve a high detection rate of the technique. 20 The surgeon's experience and technical readiness were the only statistically significant factors associated with a successful bilateral SLN biopsy in the study by Ianieri et al, in which 14 factors were analyzed. 21

In tumor-related factors, the results are also controversial. Different factors in different studies were confirmed to be statistically significant, although in others they did not show significance: lymphovascular invasion ( *p* = 0.016) and small tumor size below 2 cm ( *p* = 0.04) were associated with a high percentage of successful SLN biopsies, while nodal metastases (p = 0.03) were associated with failure of the method. 17 22 23 Other factors such as disease stage, tumor histology, degree of differentiation, and myometrial invasion did not appear to affect the success of the technique. 21 22 23 In our study, the majority of patients with successful bilateral SLN biopsy had grade 2 endometrial adenocarcinoma with tumor size more than 2cm, without lymphovascular invasion and less than 50% myometrial invasion.

In our study, SPECT/CT had the highest rate of bilateral mapping compared to planar lymphoscintigraphy and gamma probe. Similar results were reported by Elisei et al where the rate of bilateral mapping of SPECT/CT was 65%, versus gamma probe, 40%, 24 and in the study of Togami et al where the rate of bilateral detection of SPECT/CT versus planar lymphoscintigraphy was 43 versus 32%, respectively. 25

## Study Limitations

The limitations of the present study include a small sample size, and quite strict and homogeneous study protocol, so we could not analyze other potential influencing technical factors, like the effect of different radiocolloid injection techniques, radiopharmaceutical activity, imaging intervals, or surgical team. All SLN biopsies were performed by the same experienced surgeons in this field of SLN biopsy. The effect of reinjection on detection rates was not evaluated as tracer reinjection was not performed in any of our patients. It is also worth noting that patients who had extensive preoperative adhesions were not offered a SLN biopsy procedure and this factor could not have been analyzed as well.

## Conclusion

Even though the total detection rate in our study was high, the bilateral biopsy rate was 26.82%. We analyzed 16 potential predictors of unsuccessful bilateral detection of SLN in the EC but the results of our study did not confirm statistically significant factors. This may be due to the small sample size of patients we analyzed. Larger scale studies are needed to analyze the bilateral mapping of lymphatic drainage during cervical application of radiocolloid in order to determine the risk factors and improve the bilateral SLN detection in patients with EC.

This study was approved by the Ethics Committee of the Medical Faculty in Skopje (number:03-366/8).
